# Novel insights from our special issue on maternal factors during pregnancy that influence maternal, fetal and childhood outcomes

**DOI:** 10.1186/s12916-024-03278-2

**Published:** 2024-02-20

**Authors:** Stephen Tong, Katrien Benhalima, Louis Muglia, Susan Ozanne

**Affiliations:** 1 Department of Obstetrics and Gynaecology, University of Melbourne, Melbourne, VIC Australia; 2 Mercy Perinatal, Mercy Hospital for Women, Heidelberg, VIC Australia; 3 University Hospital Gasthuisberg, KU Leuven, Leuven, Belgium; 4 Burroughs Wellcome Fund, Research Triangle Park, Durham, NC USA; 5 University of Cambridge, Cambridge, UK

As guest editors of *BMC Medicine*, we issued a call in 2022 for papers that shed critical insights into factors during pregnancy that impact the health of the mother and her offspring. We are now closing submissions for this call and are delighted to announce our finalised collection: 65 new papers offering fresh insights into maternal factors during pregnancy influencing maternal, fetal and childhood outcomes.

Our opening editorial underscored the critical importance of this topic to human health. We pointed out that pregnancy is a time of remarkable physiological and psychological adaptations for both the mother and the developing fetus. Events unfolding during the months of pregnancy can reverberate for many decades on the future health of mother and child: may it be an underperforming placenta, ill-timed exposures to medications during vulnerable gestational windows or developmental tangents taken by the fetus because of variations in the genome or epigenome.

We are very pleased with the final collection, as we had hoped for a breadth of topics and varied research approaches—and we got it. Among the collection are large-scale epidemiological studies, meta-analyses, high-quality cohort studies, one randomised trial and contributions linking laboratory-generated findings (measured on biological samples)—all exploring a fascinating range of questions.

Part of the collection are insightful reviews, including an umbrella review examining risk factors for preterm birth (an adverse outcome that frequently echoes upon the lifelong health of the offspring) [1], a meta-analysis exploring potential links between breastfeeding and mental health conditions in both mother and child [2] and a fascinating narrative review on how in utero fetal programming of the metabolic syndrome may be facilitated through a failure of proper differentiation of fetal adipocytes. This incorrect differentiation limits adipocyte storage capacity, leading to lipids spill-over into the circulation and causing obesity-associated diseases [3].

There are epidemiological studies that used big data—millions of births—to generate new insights, mostly clinical. A nationwide register-based study from Sweden examined just under three million births to investigate links between maternal smoking and type 1 diabetes in the offspring [4]. Interestingly, a reduction in type 1 diabetes incidence was reported among those who smoked, though the authors do not recommend this as a public health intervention. Another population-based cohort study from Sweden reported potential harms associated with pregnant women taking proton pump inhibitors (a treatment for gastric reflux symptoms), including increased risks of preeclampsia, gestational diabetes and the birth of a small for gestational age infant [5]. Another large-scale study from Taiwan studied the potential risks for mothers of antenatal corticosteroid treatment to improve fetal health. The administration of corticosteroids was linked to an increased risk of maternal sepsis, gastrointestinal bleeding and even heart failure (though reassuringly, the absolute risk of the latter outcome remained tiny) [6].

We published one randomised trial that demonstrated the effectiveness of a mobile app, which pregnant women could use to make an appointment to consult remotely with health professionals, in lowering the risk of postpartum depressive symptoms by about one-third [7].

There are also reports describing novel applications of machine learning. Liao et al. applied artificial intelligence on readily obtainable clinical information to predict the treatments needed to achieve glycaemic control for mothers newly diagnosed with gestational diabetes [8]. Abraham and colleagues leveraged dense phenotyping on data captured on electronic health medical records to generate an algorithm to predict preterm birth risk with reasonably good accuracy, including the prediction of important subtypes of spontaneous and medically indicated preterm birth [9]. Interestingly, an artificial intelligence algorithm based on billing codes (administrative data capturing information such as specific diagnoses) outperformed algorithms based on traditional risk factors for preterm birth that clinicians currently rely upon daily.

The collection includes reports describing novel insights gleaned from the analysis of data from cohort (or case-cohort) studies collecting rich phenotypic data, ranging from physiological variables to laboratory measurements on biological samples. Among many examples, some indicated that prenatal social supports in low-risk pregnancies may somehow shape the placental epigenome (methylation differences in genes were observed that may be associated with fetal growth, energy metabolism and neurodevelopment) [10].

Another early report made links between particular bacterial species in the vaginal microbiota in pregnant women with preterm premature rupture of membranes (a high-risk condition associated with infection risk) [11] and the risk of early-onset sepsis of the neonatal soon after birth, and a large cohort study identified umbilical cord blood methylation signatures that predict rapid postnatal weight growth [12]. Interesting findings were also obtained from the Copenhagen Baby Heart Study, concluding that women with elevated body mass index birthed infants with smaller systolic and diastolic left ventricular diameters [13].

The snapshots above provide ample evidence that our collection contains a rich tapestry of diverse findings advancing this exciting area of research. We are proud to note our collection offers proof that authors from all corners of the globe are working for the same cause—to generate critical discoveries so we may better understand the maternal factors on the health and wellbeing of mother and baby and make a clinical impact.

While this collection is complete, *BMC Medicine* continues to invite submission of papers relevant to all aspects of reproductive health. As a general medical journal aiming to publish translational research with strong clinical impact, we also accept important contributions that have a strong laboratory component (including intensely mechanistic studies and in vivo animal work).

We thank all the authors of papers in this collection for publishing their research with us and *BMC Medicine* for inviting us to be guest editors. Finally, as continuing members of the editorial board, we hope those in our field will keep the manuscripts rolling in*.* This call for papers is evidence of the journal’s commitment to supporting pregnancy research. As the flagship journal of the *BMC* series, we think *BMC Medicine* is a prestigious place to publish future influential research in this area.

## Data Availability

Not applicable.
